# Crystal structure of ethyl 2-(2-{1-[*N*-(4-bromo­phen­yl)-2-oxo-2-phenyl­acetamido]-2-*tert*-butyl­amino-2-oxo­ethyl}-1*H*-pyrrol-1-yl)acetate

**DOI:** 10.1107/S2056989015023592

**Published:** 2015-12-12

**Authors:** Tetsuji Moriguchi, Venkataprasad Jalli, Suvratha Krishnamurthy, Akihiko Tsuge, Kenji Yoza

**Affiliations:** a Department of Applied Chemistry, Graduate School of Engineering, Kyushu Institute of Technology, 1-1 Sensui-cho, Tobata-ku, Kitakyushu 804-8550, Japan; b Japan Bruker AXS K.K.3-9, Moriya-cho Kanagawaku Yokohama 221-0022, Japan

**Keywords:** crystal structure, pyrrole derivative, hydrogen bonding, C—H⋯π inter­actions

## Abstract

In the title compound, C_28_H_30_BrN_3_O_5_, there is an intra­molecular N—H⋯O hydrogen bond and an intra­molecular C—H⋯O hydrogen bond, both forming *S*(9) ring motifs. The planes of the 4-bromo­phenyl ring and the phenyl ring are inclined to that of the pyrrole ring by 48.05 (12) and 77.45 (14)°, respectively, and to one another by 56.25 (12)°. In the crystal, mol­ecules are linked *via* C—H⋯O hydrogen bonds and C—H⋯π inter­actions, forming slabs parallel to (10-1).

## Related literature   

For examples of the biological and pharmacological properties of pyrrole derivatives, see: Daidone *et al.* (1990 ▸); Davis *et al.* (2008 ▸); Kaiser & Glenn (1972 ▸); Meshram *et al.* (2010 ▸).
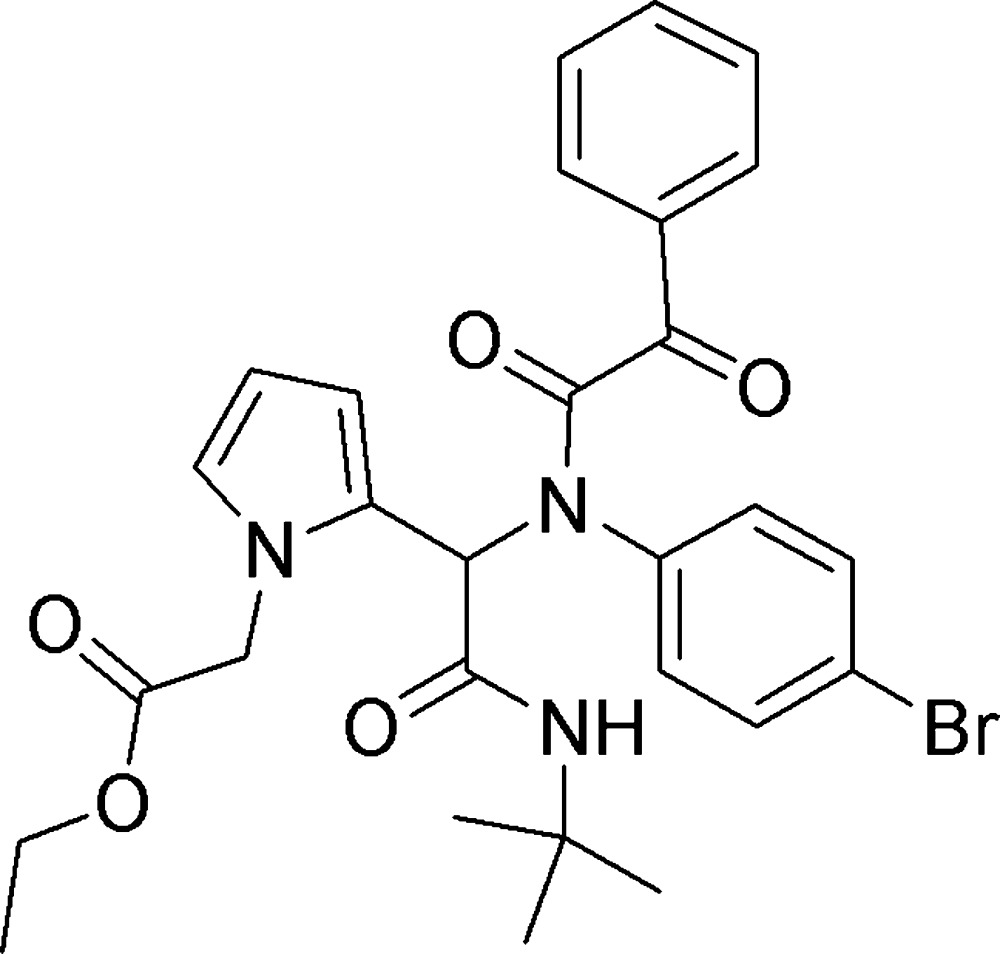


## Experimental   

### Crystal data  

C_28_H_30_BrN_3_O_5_
*M* *_r_* = 568.46
Monoclinic, 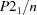

*a* = 11.656 (3) Å
*b* = 17.997 (5) Å
*c* = 13.463 (4) Å
β = 97.351 (3)°
*V* = 2801.0 (14) Å^3^
*Z* = 4
Mo *K*α radiation
μ = 1.51 mm^−1^
*T* = 120 K
0.45 × 0.45 × 0.30 mm

### Data collection  

Bruker APEXII KappaCCD diffractometer
Absorption correction: multi-scan (*SADABS*; Bruker, 2009 ▸) *T*_min_ = 0.600, *T*_max_ = 0.636
25937 measured reflections
4924 independent reflections
3479 reflections with *I* > 2σ(*I*)
*R*_int_ = 0.075

### Refinement  

*R*[*F* ^2^ > 2σ(*F* ^2^)] = 0.041
*wR*(*F* ^2^) = 0.086
*S* = 1.35
4924 reflections
338 parameters
H-atom parameters constrained
Δρ_max_ = 0.56 e Å^−3^
Δρ_min_ = −0.38 e Å^−3^

### 

Data collection: *APEX2* (Bruker, 2009 ▸); cell refinement: *SAINT* (Bruker, 2009 ▸); data reduction: *SAINT*; program(s) used to solve structure: *SHELXS97* (Sheldrick, 2008 ▸); program(s) used to refine structure: *SHELXL97* (Sheldrick, 2008 ▸); molecular graphics: *Mercury* (Macrae *et al.*, 2008 ▸); software used to prepare material for publication: *SHELXL97*.

## Supplementary Material

Crystal structure: contains datablock(s) global, I. DOI: 10.1107/S2056989015023592/su5258sup1.cif

Structure factors: contains datablock(s) I. DOI: 10.1107/S2056989015023592/su5258Isup2.hkl

Supporting information file. DOI: 10.1107/S2056989015023592/su5258Isup3.pdf

Supporting information file. DOI: 10.1107/S2056989015023592/su5258Isup4.pdf

Supporting information file. DOI: 10.1107/S2056989015023592/su5258Isup5.cml

**Fig. 1.**
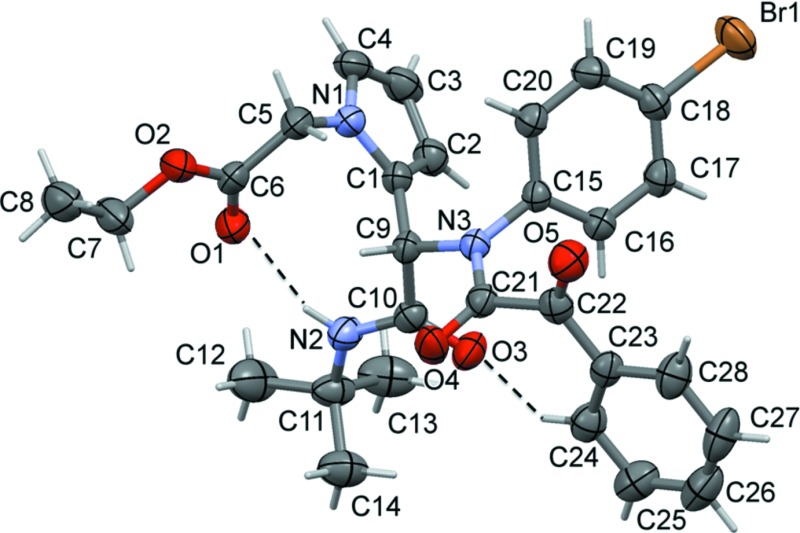
Molecular structure and atom labelling for the title compound, with displacement ellipsoids drawn at the 50% probability level.

**Fig. 2.**
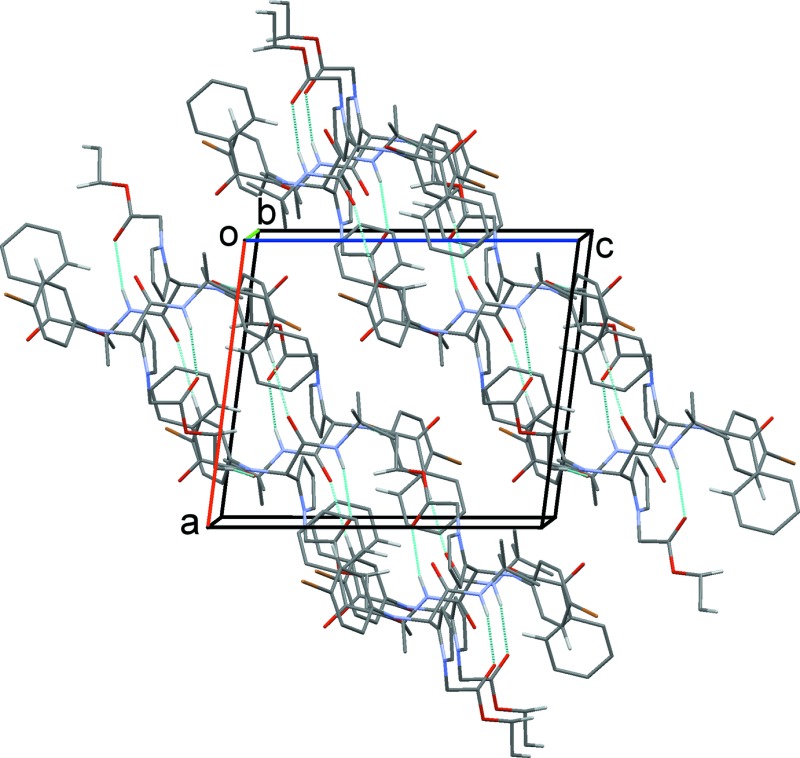
Crystal packing of the title compound, viewed along the b axis, with the hydrogen bonds shown as dashed lines (see Table 1). H atoms not involved in these reactions have been omitted for clarity.

. DOI: 10.1107/S2056989015023592/su5258fig1.tif
Mol­ecular structure and atom labelling for the title compound, with displacement ellipsoids drawn at the 50% probability level.

b . DOI: 10.1107/S2056989015023592/su5258fig2.tif
Crystal packing of the title compound, viewed along the *b* axis, with the hydrogen bonds shown as dashed lines (see Table 1). H atoms not involved in these reactions have been omitted for clarity.

**Fig. 3.**
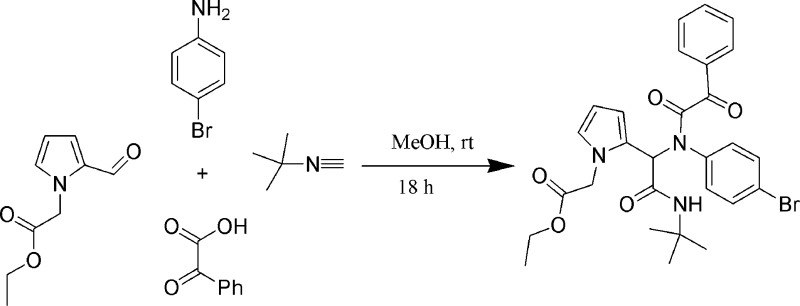
Reaction scheme for the synthesis of the title compound.

. DOI: 10.1107/S2056989015023592/su5258fig3.tif
Reaction scheme for the synthesis of the title compound.

CCDC reference: 1441330

Additional supporting information:  crystallographic information; 3D view; checkCIF report

## Figures and Tables

**Table 1:** Hydrogen-bond geometry (Å, °) *Cg*1 is the centroid of the N1/C1–C4 ring.

*D*—H⋯*A*	*D*—H	H⋯*A*	*D*⋯*A*	*D*—H⋯*A*
N2—H1⋯O1	0.86	2.13	2.970 (3)	164
C14—H24⋯O3	0.93	2.57	3.199 (3)	148
C8—H8*B*⋯O4^i^	0.96	2.55	3.432 (3)	154
C17—H17⋯O3^ii^	0.93	2.34	3.269 (3)	176
C7—H7*A*⋯*Cg*1^iii^	0.97	2.86	3.697 (3)	151

Symmetry codes: (i) 
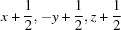
; (ii) 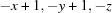; (iii) 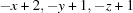.

## supplementary crystallographic information

### S1. Comments

Pyrrole and its derivatives are important classes of heterocyclic compounds because of their important biological and pharmacological properties. They have been shown to have important biological properties, such as anti­bacterial (Daidone *et al.*, 1990), anti inflammatory (Kaiser & Glenn, 1972), anti­tumor (Meshram *et al.*, 2010), and immune suppressant activities (Davis *et al.*, 2008). Pyrrole analogs are important components in naturally occurring bio molecules such as heme, chloro­phyll, vitamin B12 and pyrrole alkaloids isolated from marine sources. Highly functionalized pyrroles are found in drug molecules such as Atorvastatin, Ketorolac and Sunitinib. Thus, the elucidation of the crystal structures of pyrrole derivatives has attracted much attention. Here, we report on the crystal structure of the racemic title compound, synthesized by a four component one pot reaction, involving pyrrole-1-acetic acid-2-formyl ethyl ester, 4-bromo aniline, phenyl glyoxylic acid and *tert*-butyl isocyanide.

In the title compound, Fig. 1, there is an intra­molecular N—H···O hydrogen bonding forming an S(9) ring motif. There is also intra­molecular C—H···O hydrogen bonding which also forms an S(9) ring motif. The 4-bromo­phenyl ring and the phenyl ring are inclined to the pyrrole ring by 48.05 (12) and 77.45 (14) °, respectively, and to one another by 56.25 (12) °.

In the crystal, molecules are linked *via* C—H···O hydrogen bonds and C—H···π inter­actions forming slabs parallel to (101); see Table 1 and Fig. 2.

### S2. Synthesis and crystallization

The reaction scheme for the synthesis of the title compound is illustrated in Fig. 3. A mixture of pyrrole-1-acetic acid-2-formyl ethyl­ester (2 mmol), 4-bromo­aniline (2 mmol), phenyl­glyoxylic acid (2.2 mmol) and τ-butyl-isocyanide (2 mmol) were taken in 10 ml of MeOH and stirred at room temperature for 18 h. The volatiles were removed under reduced pressure and the pure product was isolated by column chromatography, using 30% EtOAc/Hexane, as a white coloured solid. Colourless prismatic crystals of the title compound suitable for X-ray analysis were obtained by slow evaporation of a methanol solution at room temperature. The compound crystalized in the racemic form. Spectroscopic data: LCMS: MH^+^, 568. IR (ν_max_, KBr, cm^−1^) 3144, 1740, 1730, 1725; ^1^H NMR (500 MHz, CDCl_3_, *δ*_H_) 7.99 (2 H, d), 7.57 (1 H, m), 7.44–7.47 (3 H, m), 7.14 (2 H, m), 6.8 (1 H, s), 6.61 (1 H, m), 6.14 (1 H, d), 6.09 (1 H, s), 5.99 (1 H, m), 5.65 (1 H, m), 4.74 (2 H, s), 4.29 (2 H, q), 1.36 (9 H, s), 1.33 (3 H, t).

### S3. Refinement

Crystal data, data collection and structure refinement details are summarized in Table 2. The H atoms were included in calculated positions and treated as riding atoms: N—H = 0.86 Å, C—H = 0.93 − 0.98 Å with *U*_iso_(H) = 1.5*U*_eq_(*C*-methyl) and 1.2*U*_eq_(N,*C*) for other H atoms.

### Crystal data

**Table:**

C_28_H_30_BrN_3_O_5_	*F*(000) = 1176
*M**_r_* = 568.46	*D*_x_ = 1.348 Mg m^−^^3^
Monoclinic, *P*2_1_/*n*	Mo *K*α radiation, λ = 0.71073 Å
*a* = 11.656 (3) Å	Cell parameters from 5570 reflections
*b* = 17.997 (5) Å	θ = 2.5–24.6°
*c* = 13.463 (4) Å	µ = 1.51 mm^−^^1^
β = 97.351 (3)°	*T* = 120 K
*V* = 2801.0 (14) Å^3^	Prism, colourless
*Z* = 4	0.45 × 0.45 × 0.30 mm

### Data collection

**Table:**

Bruker APEXII KappaCCD diffractometer	4924 independent reflections
Radiation source: fine focus sealed tube	3479 reflections with *I* > 2σ(*I*)
Graphite monochromator	*R*_int_ = 0.075
Detector resolution: 16.6666 pixels mm^-1^	θ_max_ = 25.0°, θ_min_ = 1.9°
ω scans	*h* = −13→13
Absorption correction: multi-scan (*SADABS*; Bruker, 2009)	*k* = −21→21
*T*_min_ = 0.600, *T*_max_ = 0.636	*l* = −15→15
25937 measured reflections	

### Refinement

**Table:**

Refinement on *F*^2^	Primary atom site location: structure-invariant direct methods
Least-squares matrix: full	Secondary atom site location: difference Fourier map
*R*[*F*^2^ > 2σ(*F*^2^)] = 0.041	Hydrogen site location: inferred from neighbouring sites
*wR*(*F*^2^) = 0.086	H-atom parameters constrained
*S* = 1.35	*w* = 1/[σ^2^(*F*_o_^2^) + (0.*P*)^2^] where *P* = (*F*_o_^2^ + 2*F*_c_^2^)/3
4924 reflections	(Δ/σ)_max_ = 0.001
338 parameters	Δρ_max_ = 0.56 e Å^−^^3^
0 restraints	Δρ_min_ = −0.38 e Å^−^^3^

### Special details

**Table:**

Geometry. All e.s.d.'s (except the e.s.d. in the dihedral angle between two l.s. planes) are estimated using the full covariance matrix. The cell e.s.d.'s are taken into account individually in the estimation of e.s.d.'s in distances, angles and torsion angles; correlations between e.s.d.'s in cell parameters are only used when they are defined by crystal symmetry. An approximate (isotropic) treatment of cell e.s.d.'s is used for estimating e.s.d.'s involving l.s. planes.
Refinement. Refinement of *F*^2^ against ALL reflections. The weighted *R*-factor *wR* and goodness of fit *S* are based on *F*^2^, conventional *R*-factors *R* are based on *F*, with *F* set to zero for negative *F*^2^. The threshold expression of *F*^2^ > σ(*F*^2^) is used only for calculating *R*-factors(gt) *etc*. and is not relevant to the choice of reflections for refinement. *R*-factors based on *F*^2^ are statistically about twice as large as those based on *F*, and *R*- factors based on ALL data will be even larger.

### Fractional atomic coordinates and isotropic or equivalent isotropic displacement parameters (Å^2^)

**Table:**

	*x*	*y*	*z*	*U*_iso_*/*U*_eq_	
Br1	0.68091 (3)	0.706481 (17)	−0.18700 (2)	0.07029 (14)	
C1	0.8525 (2)	0.53075 (13)	0.23135 (16)	0.0383 (6)	
C2	0.7777 (2)	0.58868 (14)	0.23617 (17)	0.0468 (6)	
H2	0.6979	0.5871	0.2191	0.056*	
C3	0.8433 (3)	0.65126 (15)	0.27172 (18)	0.0552 (7)	
H3	0.8149	0.6985	0.2823	0.066*	
C4	0.9549 (3)	0.62980 (15)	0.28746 (17)	0.0533 (7)	
H4	1.017	0.66	0.3114	0.064*	
C5	1.0655 (2)	0.51222 (14)	0.27953 (17)	0.0467 (7)	
H5A	1.1321	0.5449	0.2907	0.056*	
H5B	1.0727	0.483	0.2201	0.056*	
C6	1.0659 (2)	0.46056 (14)	0.36869 (17)	0.0432 (6)	
C7	1.1807 (2)	0.38299 (17)	0.4809 (2)	0.0665 (8)	
H7B	1.124	0.3436	0.4684	0.08*	
H7A	1.1667	0.4091	0.5412	0.08*	
C8	1.2984 (2)	0.35156 (16)	0.4935 (2)	0.0660 (8)	
H8A	1.3145	0.3306	0.4312	0.099*	
H8B	1.304	0.3135	0.5439	0.099*	
H8C	1.3534	0.3901	0.5137	0.099*	
C9	0.82955 (19)	0.45262 (13)	0.19813 (15)	0.0356 (6)	
H9	0.8958	0.4221	0.2256	0.043*	
C10	0.7211 (2)	0.42272 (13)	0.23900 (17)	0.0403 (6)	
C11	0.6458 (2)	0.39249 (15)	0.40046 (18)	0.0527 (7)	
C12	0.7029 (3)	0.39672 (19)	0.50841 (19)	0.0840 (11)	
H12A	0.7671	0.3629	0.5179	0.126*	
H12B	0.6477	0.3835	0.5526	0.126*	
H12C	0.7299	0.4464	0.5229	0.126*	
C13	0.5479 (3)	0.44855 (17)	0.3815 (2)	0.0737 (9)	
H13A	0.5786	0.4979	0.3912	0.111*	
H13B	0.4927	0.4398	0.4274	0.111*	
H13C	0.5106	0.4434	0.3141	0.111*	
C14	0.6037 (3)	0.31320 (15)	0.3773 (2)	0.0644 (8)	
H14A	0.5704	0.31	0.3084	0.097*	
H14B	0.5464	0.3003	0.4196	0.097*	
H14C	0.6678	0.2795	0.3893	0.097*	
C15	0.78195 (19)	0.50735 (12)	0.02338 (15)	0.0328 (5)	
C16	0.6698 (2)	0.51517 (13)	−0.02031 (16)	0.0377 (6)	
H16	0.6142	0.4808	−0.007	0.045*	
C17	0.6393 (2)	0.57413 (14)	−0.08427 (17)	0.0446 (6)	
H17	0.5637	0.5792	−0.1154	0.054*	
C18	0.7226 (2)	0.62508 (14)	−0.10092 (16)	0.0436 (6)	
C19	0.8354 (2)	0.61903 (14)	−0.05585 (17)	0.0449 (6)	
H19	0.8903	0.6545	−0.0673	0.054*	
C20	0.8652 (2)	0.55931 (13)	0.00659 (16)	0.0394 (6)	
H20	0.9408	0.554	0.0373	0.047*	
C21	0.80952 (19)	0.37438 (14)	0.05223 (17)	0.0389 (6)	
C22	0.7777 (2)	0.36402 (13)	−0.06085 (17)	0.0405 (6)	
C23	0.6628 (2)	0.33239 (13)	−0.09648 (17)	0.0387 (6)	
C24	0.5853 (2)	0.31256 (14)	−0.03116 (19)	0.0481 (7)	
H24	0.6041	0.3202	0.0373	0.058*	
C25	0.4805 (2)	0.28161 (16)	−0.0678 (2)	0.0592 (8)	
H25	0.4284	0.2686	−0.0239	0.071*	
C26	0.4525 (3)	0.26983 (16)	−0.1685 (2)	0.0647 (8)	
H26	0.3824	0.2476	−0.1926	0.078*	
C27	0.5275 (3)	0.29064 (18)	−0.2337 (2)	0.0677 (9)	
H27	0.5077	0.2836	−0.3022	0.081*	
C28	0.6322 (2)	0.32200 (16)	−0.19809 (19)	0.0563 (7)	
H28	0.6827	0.3363	−0.2427	0.068*	
N1	0.96215 (18)	0.55629 (11)	0.26258 (13)	0.0420 (5)	
N2	0.73697 (18)	0.41127 (12)	0.33778 (14)	0.0479 (6)	
H1	0.8065	0.4152	0.3676	0.058*	
N3	0.81423 (15)	0.44477 (10)	0.08742 (13)	0.0355 (5)	
O1	0.98294 (16)	0.44578 (10)	0.40912 (12)	0.0541 (5)	
O2	1.17079 (15)	0.43440 (10)	0.39589 (12)	0.0526 (5)	
O3	0.63055 (14)	0.41228 (10)	0.18436 (11)	0.0493 (4)	
O4	0.83230 (15)	0.31985 (9)	0.10509 (12)	0.0515 (5)	
O5	0.85012 (15)	0.37803 (10)	−0.11564 (12)	0.0549 (5)	

### Atomic displacement parameters (Å^2^)

**Table:**

	*U*^11^	*U*^22^	*U*^33^	*U*^12^	*U*^13^	*U*^23^
Br1	0.0898 (3)	0.04937 (19)	0.0697 (2)	0.01077 (17)	0.00284 (17)	0.02204 (15)
C1	0.0429 (16)	0.0387 (14)	0.0316 (13)	−0.0043 (13)	−0.0016 (11)	0.0022 (11)
C2	0.0557 (18)	0.0452 (16)	0.0384 (14)	−0.0001 (14)	0.0023 (12)	0.0015 (12)
C3	0.081 (2)	0.0364 (16)	0.0478 (16)	−0.0010 (16)	0.0084 (15)	0.0010 (13)
C4	0.076 (2)	0.0399 (16)	0.0429 (15)	−0.0219 (15)	0.0035 (14)	0.0025 (12)
C5	0.0469 (17)	0.0518 (17)	0.0394 (14)	−0.0152 (14)	−0.0023 (12)	0.0049 (12)
C6	0.0448 (18)	0.0438 (16)	0.0380 (14)	−0.0120 (14)	−0.0066 (13)	−0.0004 (12)
C7	0.057 (2)	0.066 (2)	0.0742 (19)	−0.0068 (16)	−0.0008 (15)	0.0323 (17)
C8	0.062 (2)	0.0514 (18)	0.082 (2)	−0.0002 (16)	−0.0024 (15)	0.0152 (16)
C9	0.0368 (15)	0.0379 (14)	0.0306 (13)	−0.0007 (11)	−0.0022 (10)	−0.0003 (10)
C10	0.0466 (17)	0.0356 (14)	0.0382 (15)	−0.0044 (12)	0.0031 (12)	−0.0032 (11)
C11	0.068 (2)	0.0486 (17)	0.0437 (15)	−0.0187 (15)	0.0173 (13)	−0.0089 (13)
C12	0.124 (3)	0.088 (3)	0.0425 (17)	−0.040 (2)	0.0212 (17)	−0.0081 (16)
C13	0.091 (3)	0.0553 (19)	0.084 (2)	−0.0089 (18)	0.0465 (18)	−0.0154 (17)
C14	0.081 (2)	0.0512 (18)	0.0652 (19)	−0.0157 (16)	0.0241 (16)	−0.0086 (14)
C15	0.0346 (15)	0.0345 (13)	0.0283 (12)	0.0014 (11)	0.0009 (10)	−0.0007 (10)
C16	0.0365 (16)	0.0388 (14)	0.0369 (13)	−0.0008 (11)	0.0021 (11)	−0.0005 (11)
C17	0.0414 (16)	0.0472 (16)	0.0431 (14)	0.0068 (13)	−0.0027 (11)	0.0003 (13)
C18	0.0522 (18)	0.0384 (15)	0.0399 (14)	0.0056 (13)	0.0043 (12)	0.0055 (12)
C19	0.0489 (18)	0.0400 (15)	0.0468 (15)	−0.0070 (13)	0.0095 (12)	0.0016 (12)
C20	0.0353 (15)	0.0421 (15)	0.0399 (14)	0.0011 (12)	0.0012 (11)	0.0001 (12)
C21	0.0340 (15)	0.0404 (15)	0.0406 (14)	0.0002 (12)	−0.0011 (11)	−0.0007 (12)
C22	0.0484 (17)	0.0330 (14)	0.0398 (14)	0.0060 (12)	0.0040 (12)	−0.0015 (11)
C23	0.0409 (16)	0.0335 (14)	0.0399 (14)	0.0058 (12)	−0.0020 (12)	−0.0076 (11)
C24	0.0498 (18)	0.0460 (17)	0.0472 (16)	0.0023 (13)	0.0007 (13)	−0.0084 (12)
C25	0.0486 (18)	0.0570 (19)	0.071 (2)	−0.0040 (15)	0.0058 (15)	−0.0129 (15)
C26	0.0471 (19)	0.059 (2)	0.083 (2)	0.0073 (15)	−0.0148 (17)	−0.0252 (17)
C27	0.056 (2)	0.089 (2)	0.0525 (18)	0.0081 (18)	−0.0132 (16)	−0.0246 (17)
C28	0.0539 (19)	0.068 (2)	0.0448 (16)	0.0059 (15)	−0.0004 (13)	−0.0126 (14)
N1	0.0485 (14)	0.0392 (12)	0.0361 (11)	−0.0104 (11)	−0.0027 (9)	0.0031 (9)
N2	0.0511 (14)	0.0567 (14)	0.0351 (12)	−0.0170 (11)	0.0024 (9)	−0.0014 (10)
N3	0.0380 (12)	0.0343 (11)	0.0326 (10)	−0.0010 (9)	−0.0020 (8)	−0.0003 (9)
O1	0.0468 (12)	0.0684 (13)	0.0452 (10)	−0.0127 (10)	−0.0012 (9)	0.0141 (9)
O2	0.0476 (12)	0.0515 (11)	0.0571 (11)	−0.0090 (9)	0.0007 (9)	0.0161 (9)
O3	0.0387 (11)	0.0630 (12)	0.0442 (10)	−0.0074 (9)	−0.0022 (8)	−0.0044 (8)
O4	0.0670 (13)	0.0364 (10)	0.0467 (10)	0.0033 (9)	−0.0097 (9)	0.0027 (8)
O5	0.0546 (12)	0.0659 (13)	0.0456 (10)	−0.0043 (10)	0.0113 (9)	−0.0064 (9)

### Geometric parameters (Å, º)

**Table:**

Br1—C18	1.893 (2)	C13—H13A	0.96
C1—C2	1.365 (3)	C13—H13B	0.96
C1—N1	1.373 (3)	C13—H13C	0.96
C1—C9	1.489 (3)	C14—H14A	0.96
C2—C3	1.410 (3)	C14—H14B	0.96
C2—H2	0.93	C14—H14C	0.96
C3—C4	1.347 (4)	C15—C16	1.370 (3)
C3—H3	0.93	C15—C20	1.387 (3)
C4—N1	1.370 (3)	C15—N3	1.439 (3)
C4—H4	0.93	C16—C17	1.384 (3)
C5—N1	1.436 (3)	C16—H16	0.93
C5—C6	1.518 (3)	C17—C18	1.375 (3)
C5—H5A	0.97	C17—H17	0.93
C5—H5B	0.97	C18—C19	1.380 (3)
C6—O1	1.198 (3)	C19—C20	1.381 (3)
C6—O2	1.318 (3)	C19—H19	0.93
C7—O2	1.465 (3)	C20—H20	0.93
C7—C8	1.473 (4)	C21—O4	1.221 (3)
C7—H7B	0.97	C21—N3	1.351 (3)
C7—H7A	0.97	C21—C22	1.532 (3)
C8—H8A	0.96	C22—O5	1.216 (3)
C8—H8B	0.96	C22—C23	1.477 (3)
C8—H8C	0.96	C23—C28	1.382 (3)
C9—N3	1.485 (3)	C23—C24	1.386 (3)
C9—C10	1.539 (3)	C24—C25	1.375 (4)
C9—H9	0.98	C24—H24	0.93
C10—O3	1.221 (3)	C25—C26	1.371 (4)
C10—N2	1.335 (3)	C25—H25	0.93
C11—N2	1.479 (3)	C26—C27	1.368 (4)
C11—C13	1.520 (4)	C26—H26	0.93
C11—C12	1.521 (4)	C27—C28	1.373 (4)
C11—C14	1.528 (3)	C27—H27	0.93
C12—H12A	0.96	C28—H28	0.93
C12—H12B	0.96	N2—H1	0.86
C12—H12C	0.96		
			
C2—C1—N1	107.7 (2)	H13B—C13—H13C	109.5
C2—C1—C9	130.1 (2)	C11—C14—H14A	109.5
N1—C1—C9	122.2 (2)	C11—C14—H14B	109.5
C1—C2—C3	107.7 (2)	H14A—C14—H14B	109.5
C1—C2—H2	126.1	C11—C14—H14C	109.5
C3—C2—H2	126.1	H14A—C14—H14C	109.5
C4—C3—C2	107.2 (3)	H14B—C14—H14C	109.5
C4—C3—H3	126.4	C16—C15—C20	120.6 (2)
C2—C3—H3	126.4	C16—C15—N3	120.0 (2)
C3—C4—N1	109.1 (2)	C20—C15—N3	119.4 (2)
C3—C4—H4	125.5	C15—C16—C17	120.1 (2)
N1—C4—H4	125.5	C15—C16—H16	120.0
N1—C5—C6	112.4 (2)	C17—C16—H16	120.0
N1—C5—H5A	109.1	C18—C17—C16	118.8 (2)
C6—C5—H5A	109.1	C18—C17—H17	120.6
N1—C5—H5B	109.1	C16—C17—H17	120.6
C6—C5—H5B	109.1	C17—C18—C19	121.9 (2)
H5A—C5—H5B	107.9	C17—C18—Br1	118.99 (19)
O1—C6—O2	124.6 (2)	C19—C18—Br1	119.08 (19)
O1—C6—C5	125.1 (2)	C18—C19—C20	118.7 (2)
O2—C6—C5	110.3 (2)	C18—C19—H19	120.7
O2—C7—C8	108.2 (2)	C20—C19—H19	120.7
O2—C7—H7B	110.1	C19—C20—C15	119.8 (2)
C8—C7—H7B	110.1	C19—C20—H20	120.1
O2—C7—H7A	110.1	C15—C20—H20	120.1
C8—C7—H7A	110.1	O4—C21—N3	123.6 (2)
H7B—C7—H7A	108.4	O4—C21—C22	119.2 (2)
C7—C8—H8A	109.5	N3—C21—C22	117.2 (2)
C7—C8—H8B	109.5	O5—C22—C23	123.6 (2)
H8A—C8—H8B	109.5	O5—C22—C21	118.6 (2)
C7—C8—H8C	109.5	C23—C22—C21	117.6 (2)
H8A—C8—H8C	109.5	C28—C23—C24	119.1 (2)
H8B—C8—H8C	109.5	C28—C23—C22	118.8 (2)
N3—C9—C1	112.66 (18)	C24—C23—C22	122.1 (2)
N3—C9—C10	109.08 (17)	C25—C24—C23	119.9 (2)
C1—C9—C10	110.28 (18)	C25—C24—H24	120.1
N3—C9—H9	108.2	C23—C24—H24	120.1
C1—C9—H9	108.2	C26—C25—C24	120.4 (3)
C10—C9—H9	108.2	C26—C25—H25	119.8
O3—C10—N2	125.1 (2)	C24—C25—H25	119.8
O3—C10—C9	121.6 (2)	C27—C26—C25	120.1 (3)
N2—C10—C9	113.2 (2)	C27—C26—H26	119.9
N2—C11—C13	109.4 (2)	C25—C26—H26	119.9
N2—C11—C12	106.0 (2)	C26—C27—C28	120.0 (3)
C13—C11—C12	110.7 (2)	C26—C27—H27	120.0
N2—C11—C14	109.4 (2)	C28—C27—H27	120.0
C13—C11—C14	111.8 (2)	C27—C28—C23	120.5 (3)
C12—C11—C14	109.4 (2)	C27—C28—H28	119.8
C11—C12—H12A	109.5	C23—C28—H28	119.8
C11—C12—H12B	109.5	C4—N1—C1	108.3 (2)
H12A—C12—H12B	109.5	C4—N1—C5	124.8 (2)
C11—C12—H12C	109.5	C1—N1—C5	126.4 (2)
H12A—C12—H12C	109.5	C10—N2—C11	125.8 (2)
H12B—C12—H12C	109.5	C10—N2—H1	117.1
C11—C13—H13A	109.5	C11—N2—H1	117.1
C11—C13—H13B	109.5	C21—N3—C15	121.87 (18)
H13A—C13—H13B	109.5	C21—N3—C9	115.81 (18)
C11—C13—H13C	109.5	C15—N3—C9	121.04 (17)
H13A—C13—H13C	109.5	C6—O2—C7	114.86 (19)
			
N1—C1—C2—C3	−0.3 (3)	C23—C24—C25—C26	0.4 (4)
C9—C1—C2—C3	−179.3 (2)	C24—C25—C26—C27	−1.7 (4)
C1—C2—C3—C4	−0.1 (3)	C25—C26—C27—C28	1.3 (5)
C2—C3—C4—N1	0.4 (3)	C26—C27—C28—C23	0.4 (4)
N1—C5—C6—O1	14.3 (3)	C24—C23—C28—C27	−1.7 (4)
N1—C5—C6—O2	−165.40 (19)	C22—C23—C28—C27	178.0 (3)
C2—C1—C9—N3	79.9 (3)	C3—C4—N1—C1	−0.6 (3)
N1—C1—C9—N3	−99.0 (2)	C3—C4—N1—C5	−173.0 (2)
C2—C1—C9—C10	−42.2 (3)	C2—C1—N1—C4	0.5 (2)
N1—C1—C9—C10	138.9 (2)	C9—C1—N1—C4	179.64 (19)
N3—C9—C10—O3	−15.8 (3)	C2—C1—N1—C5	172.8 (2)
C1—C9—C10—O3	108.4 (2)	C9—C1—N1—C5	−8.0 (3)
N3—C9—C10—N2	165.23 (19)	C6—C5—N1—C4	103.3 (3)
C1—C9—C10—N2	−70.6 (3)	C6—C5—N1—C1	−67.8 (3)
C20—C15—C16—C17	1.9 (3)	O3—C10—N2—C11	−7.5 (4)
N3—C15—C16—C17	−178.1 (2)	C9—C10—N2—C11	171.4 (2)
C15—C16—C17—C18	−1.5 (3)	C13—C11—N2—C10	−52.2 (3)
C16—C17—C18—C19	0.0 (4)	C12—C11—N2—C10	−171.6 (2)
C16—C17—C18—Br1	−179.23 (17)	C14—C11—N2—C10	70.6 (3)
C17—C18—C19—C20	1.0 (4)	O4—C21—N3—C15	−177.3 (2)
Br1—C18—C19—C20	−179.78 (17)	C22—C21—N3—C15	4.8 (3)
C18—C19—C20—C15	−0.5 (3)	O4—C21—N3—C9	−10.1 (3)
C16—C15—C20—C19	−0.9 (3)	C22—C21—N3—C9	172.03 (19)
N3—C15—C20—C19	179.1 (2)	C16—C15—N3—C21	64.9 (3)
O4—C21—C22—O5	−102.8 (3)	C20—C15—N3—C21	−115.1 (2)
N3—C21—C22—O5	75.1 (3)	C16—C15—N3—C9	−101.6 (2)
O4—C21—C22—C23	73.0 (3)	C20—C15—N3—C9	78.4 (3)
N3—C21—C22—C23	−109.1 (2)	C1—C9—N3—C21	171.0 (2)
O5—C22—C23—C28	−4.0 (4)	C10—C9—N3—C21	−66.2 (2)
C21—C22—C23—C28	−179.6 (2)	C1—C9—N3—C15	−21.7 (3)
O5—C22—C23—C24	175.7 (2)	C10—C9—N3—C15	101.1 (2)
C21—C22—C23—C24	0.1 (3)	O1—C6—O2—C7	1.0 (3)
C28—C23—C24—C25	1.3 (4)	C5—C6—O2—C7	−179.3 (2)
C22—C23—C24—C25	−178.4 (2)	C8—C7—O2—C6	172.8 (2)

### Hydrogen-bond geometry (Å, º)

Cg1 is the centroid of the N1/C1–C4 ring.

**Table:**

*D*—H···*A*	*D*—H	H···*A*	*D*···*A*	*D*—H···*A*
N2—H1···O1	0.86	2.13	2.970 (3)	164
C14—H24···O3	0.93	2.57	3.199 (3)	148
C8—H8*B*···O4^i^	0.96	2.55	3.432 (3)	154
C17—H17···O3^ii^	0.93	2.34	3.269 (3)	176
C7—H7*A*···*Cg*1^iii^	0.97	2.86	3.697 (3)	151

Symmetry codes: (i) *x*+1/2, −*y*+1/2, *z*+1/2; (ii) −*x*+1, −*y*+1, −*z*; (iii) −*x*+2, −*y*+1, −*z*+1.

## References

[bb1] Bruker (2009). *APEX2*, *SAINT* and *SADABS*. Bruker AXS Inc., Madison, Wisconsin, USA.

[bb2] Daidone, G., Maggio, B. & Schillaci, D. (1990). *Pharmazie*, **45**, 441–442. 2402534

[bb3] Davis, F. A., Bowen, K., Xu, H. & Velvadapu, V. (2008). *Tetrahedron*, **64**, 4174–4182. 10.1016/j.tet.2008.02.102 PMC2391011 19421309

[bb4] Kaiser, D. G. & Glenn, E. M. (1972). *J. Pharm. Sci.* **61**, 1908–1911. 10.1002/jps.2600611205 4638096

[bb5] Macrae, C. F., Bruno, I. J., Chisholm, J. A., Edgington, P. R., McCabe, P., Pidcock, E., Rodriguez-Monge, L., Taylor, R., van de Streek, J. & Wood, P. A. (2008). *J. Appl. Cryst.* **41**, 466–470.

[bb6] Meshram, H. M., Prasad, B. R. V. & Kumar, D. A. (2010). *Tetrahedron Lett.* **51**, 3477–3480.

[bb7] Sheldrick, G. M. (2008). *Acta Cryst.* A**64**, 112–122. 10.1107/S0108767307043930 18156677

