# Supplementary material for: Crystal structure of ethyl 2-(2-{1-[N-(4-bromo­phen­yl)-2-oxo-2-phenyl­acetamido]-2-tert-butyl­amino-2-oxo­ethyl}-1H-pyrrol-1-yl)acetate
Source: Acta Crystallogr E Crystallogr Commun. 2015 Dec 12;71(Pt 12):o1049–50. doi: 10.1107/S2056989015023592 (PMC4719973; doi:10.1107/S2056989015023592)
Supplement: Supplementary file 4 [file e-71-o1049-Isup4.pdf]

checkCIF () running

Checking for embedded fcf data in CIF ...
No extractable fcf data in found in CIF

checkCIF/PLATON (full publication check)

You have not supplied any structure factors. As a result the full set of tests cannot be run.

THIS REPORT IS FOR GUIDANCE ONLY. IF USED AS PART OF A REVIEW PROCEDURE FOR PUBLICATION, IT SHOULD NOT REPLACE THE EXPERTISE OF AN EXPERIENCED CRYSTALLOGRAPHIC REFEREE.You have not supplied any structure factors. As a result the full set of tests cannot be run.

No syntax errors found.
Please wait while processing ....

CIF dictionary
Interpreting this report

Datablock: I

Bond precision: C-C = 0.0034 A Wavelength=0.71073

Cell:	a=11.656(3)	b=17.997(5)	c=13.463(4)
	alpha=90	beta=97.351(3)	gamma=90
Temperature:	120 K		

	Calculated	Reported
Volume	2801.0(13)	2801.0(14)
Space group	P 21/n	P 1 21/n 1
Hall group	-P 2yn	-P 2yn
Moiety formula	C28 H30 Br N3 O5	C28 H30 Br N3 O5
Sum formula	C28 H30 Br N3 O5	C28 H30 Br N3 O5
Mr	568.45	568.46
Dx,g cm-3	1.348	1.348
Z	4	4
Mu (mm-1)	1.508	1.508
F000	1176.0	1176.0
F000'	1175.34	
h,k,lmax	13,21,16	13,21,15
Nref	4946	4924
Tmin,Tmax	0.512,0.636	0.600,0.636
Tmin'	0.502	

Correction method= # Reported T Limits: Tmin=0.600 Tmax=0.636 AbsCorr = EMPIRICAL

Data completeness= 0.996 Theta(max)= 25.030

R(reflections)= 0.0406(3479) wR2(reflections)= 0.0862(4924)

S = 1.352 Npar= 338

The following ALERTS were generated. Each ALERT has the format
test-name_ALERT_alert-type_alert-level.
Click on the hyperlinks for more details of the test.

Alert level C

PLAT369_ALERT_2_C Long C(sp2)-C(sp2) Bond C21 - C22 .. 1.53 Ang.

Alert level G

PLAT005_ALERT_5_G No Embedded Refinement Details found in the CIF Please Do !

PLAT007_ALERT_5_G Number of Unrefined Donor-H Atoms .... 1 Report
PLAT793_ALERT_4_G The Model has Chirality at C9 (Centro SPGR) S Verify

0 **ALERT level A** = Most likely a serious problem - resolve or explain
0 **ALERT level B** = A potentially serious problem, consider carefully
1 **ALERT level C** = Check. Ensure it is not caused by an omission or oversight
3 **ALERT level G** = General information/check it is not something unexpected

0 ALERT type 1 CIF construction/syntax error, inconsistent or missing data
1 ALERT type 2 Indicator that the structure model may be wrong or deficient
0 ALERT type 3 Indicator that the structure quality may be low
1 ALERT type 4 Improvement, methodology, query or suggestion
2 ALERT type 5 Informative message, check

Publication of your CIF
You should attempt to resolve as many as possible of the alerts in all categories. Often the minor alerts point to easily fixed oversights, errors and omissions in your CIF or refinement strategy, so attention to these fine details can be worthwhile. In order to resolve some of the more serious problems it may be necessary to carry out additional measurements or structure refinements. However, the nature of your study may justify the reported deviations from journal submission requirements and the more serious of these should be commented upon in the discussion or experimental section of a paper or in the "special_details" fields of the CIF. *checkCIF* was carefully designed to identify outliers and unusual parameters, but every test has its limitations and alerts that are not important in a particular case may appear. Conversely, the absence of alerts does not guarantee there are no aspects of the results needing attention. It is up to the individual to critically assess their own results and, if necessary, seek expert advice.
If you wish to submit your CIF for publication in Acta Crystallographica Section C or E, you should upload your CIF via the web. If your CIF is to form part of a submission to another IUCr journal, you will be asked, either during electronic submission or by the Co-editor handling your paper, to upload your CIF via our web site.

PLATON version of 19/11/2015; check.def file version of 17/11/2015

Datablock I - ellipsoid plot

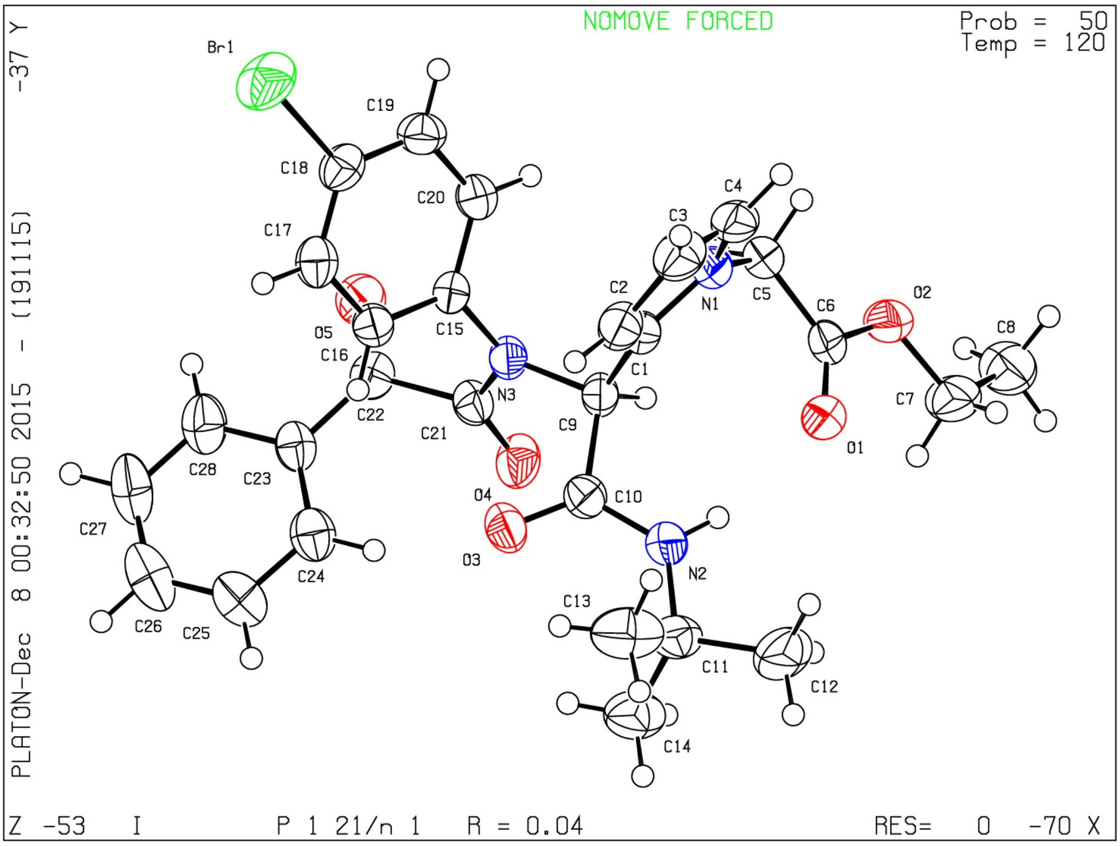

Download CIF editor (publCIF) from the IUCr
Download CIF editor (enCIFer) from the CCDC
Test a new CIF entry
