# Supplementary figures and images for: Crystal structure of ethyl 2-(2-{1-[N-(4-bromo­phen­yl)-2-oxo-2-phenyl­acetamido]-2-tert-butyl­amino-2-oxo­ethyl}-1H-pyrrol-1-yl)acetate
Source: Acta Crystallogr E Crystallogr Commun. 2015 Dec 12;71(Pt 12):o1049–50. doi: 10.1107/S2056989015023592 (PMC4719973; doi:10.1107/S2056989015023592)

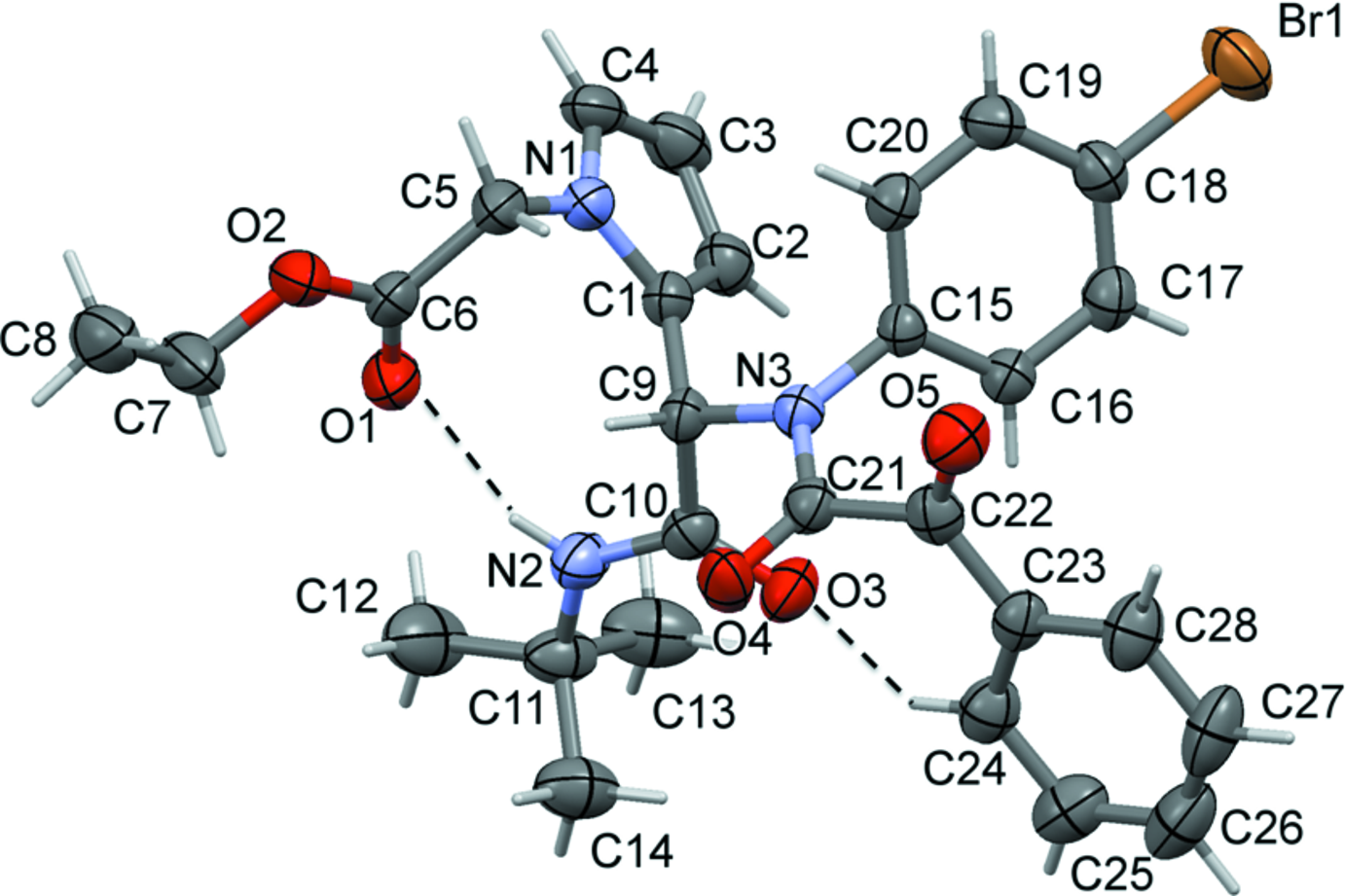

Supplement: Supplementary file 6 [file e-71-o1049-fig1.tif]

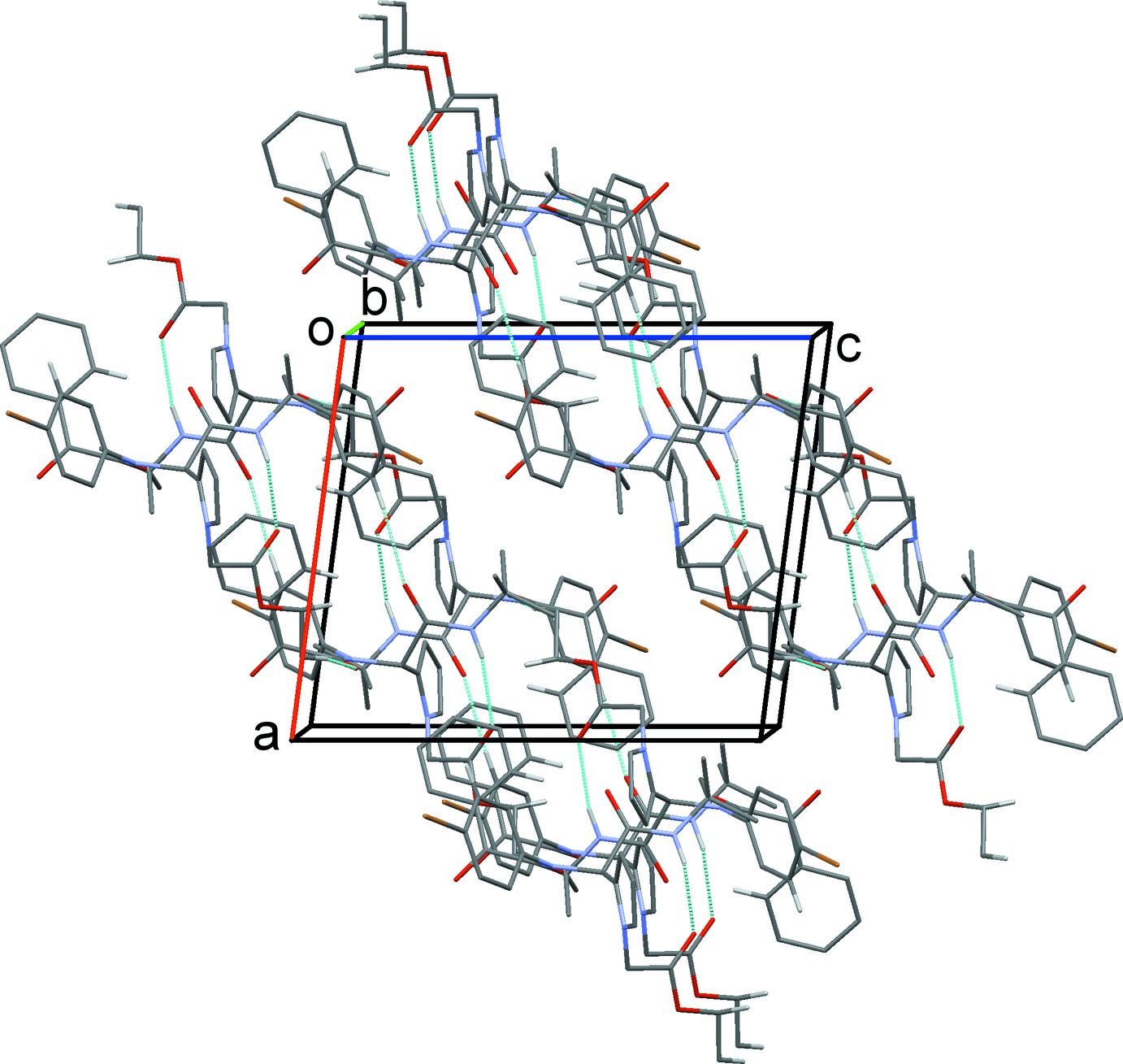

Supplement: Supplementary file 7 [file e-71-o1049-fig2.tif]
